# Physiological and Oxidative Stress Responses of Lettuce to Cleomside A: A Thiohydroximate, as a New Allelochemical from *Cleome arabica* L.

**DOI:** 10.3390/molecules25194461

**Published:** 2020-09-28

**Authors:** Afef Ladhari, Anna Andolfi, Marina DellaGreca

**Affiliations:** 1 Laboratoire GREEN-TEAM (LR17AGR01), Institut National Agronomique de Tunisie (INAT), Universite de Carthage, 43 Avenue Charles Nicolle, Tunis 1082, Tunisia; 2 Dipartimento di Scienze Chimiche, Università Federico II, Complesso Universitario Monte S. Angelo, via Cintia, 4, 80126 Napoli, Italy; andolfi@unina.it (A.A.); dellagre@unina.it (M.D.); 3 BAT Center-Interuniversity Center for Studies on Bioinspired Agro-Environmental Technology, University of Napoli ‘Federico II’, 80138 Naples, Italy

**Keywords:** allelochemicals, *Cleome arabica*, thiohydroximate, oxidative damage, membrane integrity

## Abstract

The inclination toward natural products have led the onset for the discovery of new bioactive metabolites that could be targeted for specific therapeutic or agronomic applications. This study aimed to isolate bioactive compounds from *Cleome arabica* L., and subsequently determine the unexplored mechanism of action of the newly identified compounds on *Lactuca sativa* L. Chemical investigation of the ethyl acetate fraction of methanolic silique extract of *C. arabica* afforded seven secondary metabolites belonging to different classes such as flavonoids, triterpene, and a new thiohydroximate derivative, named cleomside A. Among phytotoxic assays, the growth of lettuce was totally inhibited by cleomside A compared to the other identified compounds. This effect was associated with the increased levels of electrolyte leakage, malondialdehyde, and hydrogen peroxide indicating disruption of membrane integrity and induction of oxidative stress. Activities of the antioxidant enzymes SOD, CAT, and APX were also elevated, thereby demonstrating the enhanced generation of reactive oxygen species upon identified allelochemical exposure. Thus, the changes caused by cleomside A described herein can contribute to better understanding the allelochemical actions of thiohydroximate and the potential use of these substances in the production of natural herbicides compared to the other identified flavonoids and triterpene.

## 1. Introduction

In the agricultural field, many invasive species have demonstrated resistance to commercially available herbicides, which cause about 32% of yield reduction, and therefore lead to huge economic losses [1]. Meanwhile, the production of synthetic herbicides was declined since the last two decades due to the absence of the discovery of a new target site of action [2]. In the last few years, many efforts have been devoted in order to discover an alternative natural phytotoxic product that might help in resolving the dependency on synthetic herbicides in a safety way considering the environment and human health [2,3]. Generally, many phytotoxic products are isolated and identified from different plant tissues, and to shed light on these phytotoxic compounds, it is important to discover new natural components with new target site that could offer interesting templates for potential pharmaceutical and agricultural applications [4]. 

*Cleome arabica* L. (genus Cleome) an annual herb in the family of Capparidaceae, is abundant in sandy environments, and the gravel, and stony grounds in arid Tunisia. This plant possesses important pharmaceutical, economic, and ecologic values. In addition, according to our previous studies [3], different tissues of *C. arabica* possessed potent phytotoxic effect mainly by the silique, that might be an alternative to develop natural herbicides for a sustainable agriculture. Besides that, it was noted that their dried parts are markedly browsed and appreciated by animals but are generally denied when it is fresh plant. This repulsion seems to be explained by the presence of certain repellant toxic compounds. Over the years, limited phytochemical investigation of *C. arabica* led to the isolation of phenolic compounds, alkaloids [5], dammarane triterpene [3,4,5,6], cleomin [7], new steroid derivatives [8], and glucosylated and rhamnosylated flavonols [9]. In fact, glucosinolates are the major organoleptic and bioactive constituents of the Capparidaceae, Brassicaceae and a few other related plant families [10,11]. Glucosinolates compounds have long been known for their fungicidal, bactericidal, nematocidal and allelopathic properties, and have recently attracted attention researchers because of their cancer chemoprotective attributes [12]. Although, scientists highlighted the negative aspects of glucosinolate compounds because of the prevalence of certain “antinutritional” or goitrogenic glucosinolates degradation products, and ultimately affect the productivity of animals [13]. Since there are very few studies on the identified active compounds form *C. arabica*, it is crucial to continue discovering new bioactive allelochemicals from the most active plant organ [3] that could promote new target site of action. Correlating the phytotoxicity assays with the physiological effect could understand deeper the mechanism of action of the active compound of *C. arabica* [14]. Actually, there are no work in the literature that correlate the main phytotoxic effects of *C. arabica* compounds with cellular events related to physiological, cytological, and antioxidant enzymes activities. Subsequent studies on the mode of action of the allelochemical stress could cause an oxidative damage, proved by membrane lipid peroxidation as well as an increase in antioxidant enzymes with a marked enhancement of the reactive oxygen species (ROS) [15]. Based on this evidence, we intended to investigate more about the effects of allelochemicals produced by *C. arabica* on the antioxidant system, and if an imbalance in early germination stages could be related with the root inhibition, as well as to answer if this stress induced either defense or cell death responses. For these reasons, the present study aimed to identify new active allelochemicals from siliquae as the active organ of *C. arabica*. Then, we analyzed the effects of allelopathic stress caused by *C. arabica* extract and its identified compounds on *Lactuca sativa* L. at the physiological and cytological levels, particularly on activities of the antioxidant enzymes such as catalase (CAT), ascorbate peroxidase (APX), and superoxide dismutase (SOD). In addition, the hydrogen peroxide amount (H_2_O_2_) and membrane damage as lipid peroxidation were determined.

## 2. Results and Discussion

### 2.1. Structural Elucidation of Secondary Metabolites

**Figure 1:**
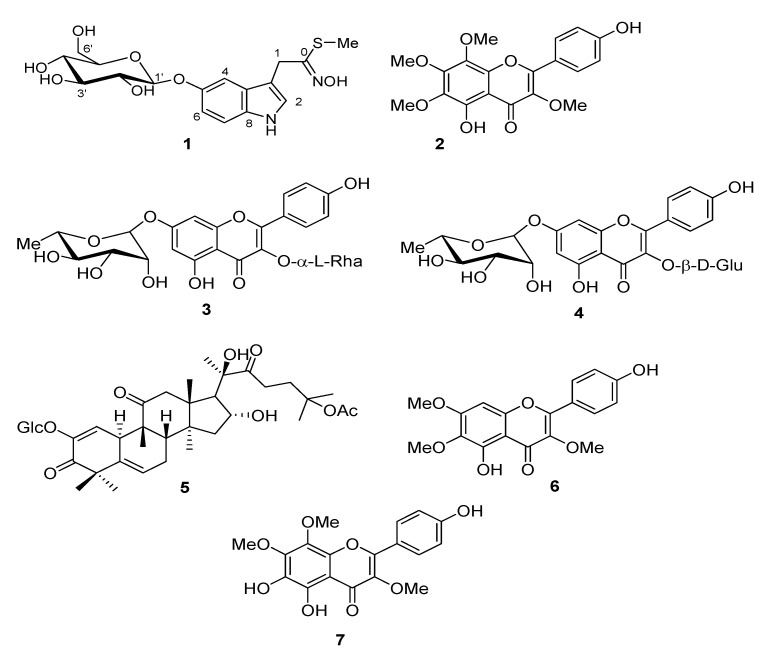
Structures of compounds **1**–**7** isolated from the silique of *Cleome arabica.*

Repeated chromatographic purification of methanol extract of *C. arabica* yield to isolation of an unusual indole derivative (compound **1**) whose structure was assigned by extensive spectroscopic analysis and six know compounds (**2**–**7**, Figure 1). Compound **1** (Figure 1) was obtained as light-yellow oil and showed bands at λ_max_ 207, 268 and 304 nm in UV spectrum. The structure of this compound was determined on the basis of spectroscopic data (Supplementary Materials Figures S1–S7). The molecular formula C_17_H_22_N_2_O_7_S was suggested by positive molecular peak at *m/z* 399.1218 [M + H]^+^ in its HRESI-MS (Figure S8) and ^13^C NMR spectra corresponding to eight degrees of unsaturation. The ^1^H NMR (Table 1) displayed a methyl signal at δ H 2.24 as singlet, two methylene signals, of which one as two double doublets at δ H 3.92 (*J* = 13.5, 10.2 Hz) and 3.75 (*J* = 12.0, 5.4 Hz) and one as two doublets at δ H 4.78 and 4.50 (*J* = 14.4 Hz), and five methine signals, of which one as doublet at δ H 5.10 (*J* = 7.1 Hz) and four as multiplets in the δ H 3.61–3.40 range in the aliphatic region. The ^1^H NMR (Table 1) also displayed four signals at δ H 7.10, 7.02 (×2) and 6.80 in the aromatic region. The ^13^C NMR (DEPT, Table 1) spectrum indicated that compound **1** possesses 16 carbon signals, involving one methyl, two methylenes, nine methines (including four aromatics), and five quaternary carbons.

**Figure 2:**
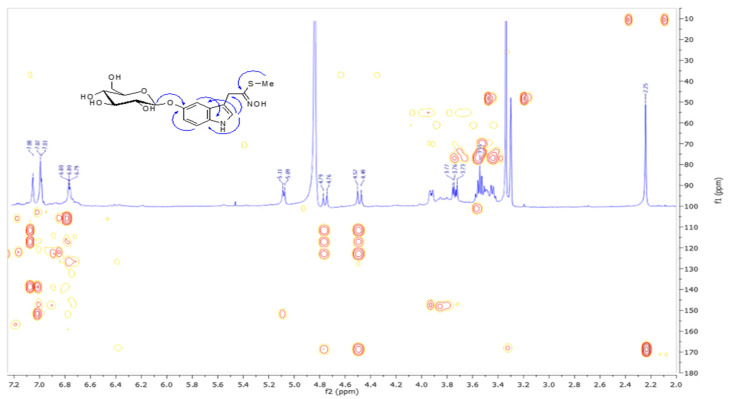
HMBC spectrum of cleomside A (**1**).

The protons were assigned to the corresponding carbons by an HSQC experiment (Table 1). The COSY spectrum indicated the presence of a hexose unit, which on the basis of the ^1^H and ^13^C chemical shifts and coupling constants of anomeric proton was identified as β-*O*-glucopyranoside [16]. The aromatic portion was identified as indolic unit on the basis of ^1^H and ^13^C chemical shifts and long-range heterocorrelations observed in the HMBC spectrum (Figure 2). The most important correlations observed for the indole were: the protons at δ 7.10 and 7.02 attributed to the H-2, H-4, and H-7 respectively with the C-8 carbon at δ 140.5, and the first proton was also correlated to the sp^3^ carbon at δ 39.0 (C-1), C-3 (δ 113.5), and C-9 (δ 119.0) carbons; the H-7 proton was also correlated to the C-5 (δ 153.5), carbon as well as the H-6 proton (δ 6.80) gave cross peaks with the C-4, C-7 (δ 123.9) and C-8 carbons. In the HMBC spectrum heterocorrelation of the anomeric proton at δ 5.10 with the C-5 carbon was observed indicating that the compound was glucosylated on indole. Beside the already reported correlations, the H-1 and methyl protons were correlated to the C-0 (δ 171.0) defining a methyl *N*-hydroxyimidothioate group. NOESY spectrum evidenced correlation between the anomeric proton (δ 5.10) and aromatic proton at δ6.80 and H-5′ (δ 3.61). All these data led to the structure of methyl *N*-hydroxy-2-(5′-*O*-β-d-glucopyranosyl)-1H-indol-3-yl)ethanimidothioate (**1**) named cleomside A.

The flavonoid nature of compounds **2**, **3**, **4**, **6** and **7** (Figure 1) was inferred from the analysis of spectral data, especially ^1^H- and ^13^C- spectra, that allowed us to elucidate their structures. 5,4′-Dihydroxy-3,6,7,8-tetramethoxyflavone (calycopterin, **2**), kaempferol 3,7-*O*-α-L-dirhamnopyranoside (kaempferitrin, **3**), kaempferol 3-O-β-D-glucopyranoside-7-*O*-α-L-rhamnopyranoside (**4**), 5,4′-dihydroxy-3,6,7 -trimethoxyflavone (penduletin, **6**), 5,6,4′-trihydroxy-3,7,8-trimethoxyflavone (**7**), were identified by comparison of their spectral data with those reported in literature [17,18].

Compound **5** was identified as a triterpene belong to cucurbitane group. Inspection of ^1^H- and ^13^C-NMR spectra revealed that this compound possess a monosaccaride unit. The ^13^C-NMR spectrum of **5** displayed 36 carbon signals, of which 30 were attributed to the triterpenoid moiety and six to the sugar moiety. Thus, compound was identified as 25-*O*-acetylbryoamaride (Figure 1) by comparison of its NMR data with values reported in the literature [19].

### 2.2. Phytotoxicity Assays

**Figure 3:**
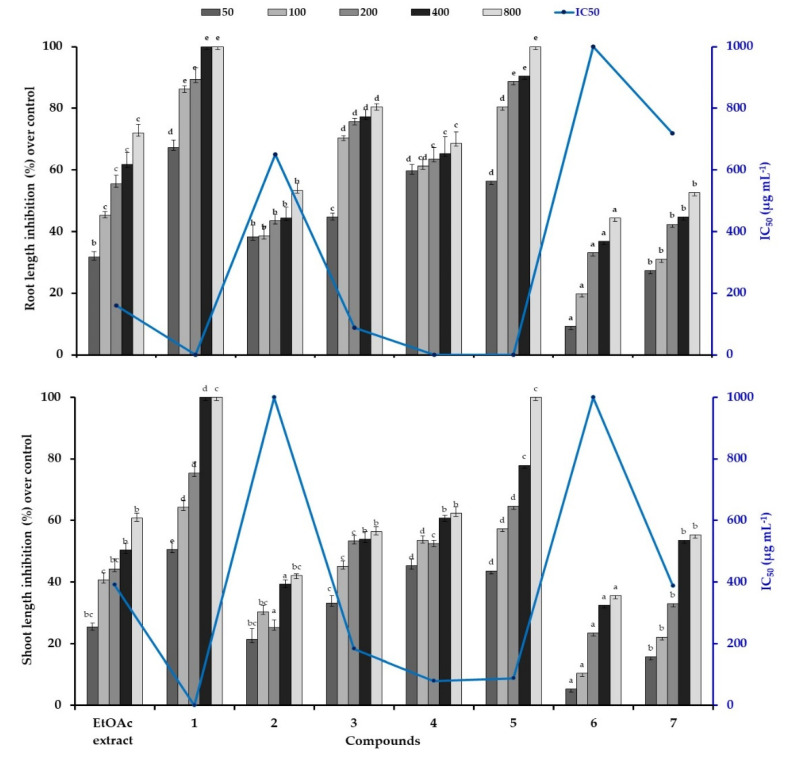
Phytotoxic effect of silique ethyl acetate extract of *C. arabica* and its identified compounds on root/shoot length of lettuce at different concentration (50, 100, 200, 400, 600, and 800 µg mL^−1^). The bars on each column show standard error. Values (*N* = 3 ± S.E.). Different letters in columns indicate significant differences among treatments at *p* < 0.05 (LSD test).

The conducted analysis revealed that all the treatments possesses a significant phytotoxic effect on lettuce germination and growth. The inhibitory magnitude was varied according to the concentration of the tested samples (Table 2; Figure 3). In fact, all the treatments had similar frequency of germinated lettuce compared to the control, except those exposed to compounds **1** (cleomoside A) and **5** (25-*O*-acetylbryoamaride), which had an inhibition of 100% at the highest concentration (Table 2). The significant effect of compound **1** was markedly depicted even at the lowest concentration by an inhibition of 73.7% compared to the compound **5** (34.7%). In addition, the compound **1** and **5** revealed the lowest IC_50_ values, which were estimated less than 50 µg mL^−1^ and 91.4 µg mL^−1^, respectively. While, the EtOAc extract and the other tested compounds possess IC_50_ values greater than 800 µg mL^−1^.

Similarly, the compounds **1** and **5** exerted the highest phytotoxic effect on lettuce growth indicated by a total inhibition from 400 and 800 µg mL^−1^, respectively (Figure 3). In all cases, the root length was more sensitive than shoot, particularly in the presence of EtOAc extract, the compounds **3** and **4** in which they revealed an average inhibition of 73.6% and 59.7% for root and shoot lengths, respectively, at the highest concentration. While, the other compounds exhibited moderate inhibition of 47.2% on lettuce seedling growth. Thus, the highest phytotoxic effect of the active compounds was displayed by the lowest IC_50_ values, which was estimated under 50 µg mL^−1^ in the presence of the compound **1** for root and shoot lengths. Similarly, the compounds **4** and **5** possess the lowest IC_50_ values (under 50 µg mL^−1^) for root lengths and was about 83.8 µg mL^−1^ for shoot lengths. The EtOAc extract and the compound **7** revealed IC_50_ value of 389 µg mL^−1^, while the compound **3** possess a value of 183 µg mL^−1^. However, the highest IC_50_ value was estimated about more than 800 µg mL^−1^ by the compounds **6** and **2** (Figure 3). 

Generally, in response to various tested flavonoids, germination was slightly affected, whereas significant difference was observed against radical elongation. It could be explained by the entry of water through the integument during the germination process produced the entry of bioactive compounds, by mass flow, which began their physiological activities in the next phase of root growth [20]. While, the identified triterpene (compound **5**) and thiohydroximate (compound **1**) induced the highest phytotoxic effect even at the lowest concentration While, the identified triterpene (compound **5**) and thiohydroximate (compound **1**) induced the highest phytotoxic effect even at the lowest concentration. To our knowledge there is no study considering the phytotoxic effect of these identified compounds neither on germination nor on seedling growth. Our results confirmed earlier studies by showing that some thiohydroximate derivatives can be phytotoxic at high concentrations. It was noted that thiohydroximates occur most commonly as precursors of glucosinolates, important plant natural products [21]. Besides that, the knowledge of flavonoids involved in plant–plant interactions and their mechanisms of action are poor and, moreover, the structural characteristics required for these biological activities are scarcely known. The marked phytotoxic effect of terpenoids compared to flavonoids was confirmed by Ladhari et al. [3], which they revealed that the identified terpenoid in *C. arabica* silique induced an important inhibition of 70% compared to calycopterin (inhibition of 33%) on lettuce growth at 0.06 g L^−1^. Some flavonoids appear to act primarily as germination and cell growth inhibitors, possibly through interference with the energy transfer system within the cell [22]. Flavones have been shown to interfere with ATP formation in plant mitochondria [23]. Modifications of plant growth and development in response to exposure to allelochemicals may reflect alterations in the molecular biology of cells, their ultrastructure, or their biochemical and physiological processes, which have been the focus of some reviews [24]. Often, allelochemicals effects are reported descriptively, missing integrative models to explain how these molecules could affect cellular processes.

### 2.3. Mitotic Index in Lettuce Roots

**Figure 4:**
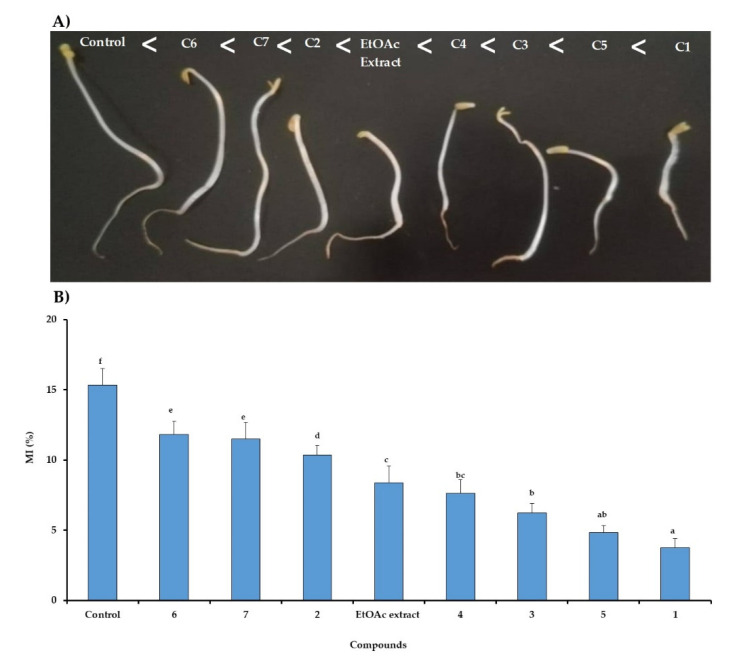
Relationship between mitotic index during 48 h (**A**) and morphological responses in 7 days (**B**) to EtOAc extract of *C. arabica* and its identified compounds (C1-C7) at 50 µg mL^−1^ on seedling growth of lettuce. The bars on each column show standard error. Values (*N* = 3 ± S.E.). Different letters in columns indicate significant differences among treatments at *p* < 0.05 (LSD test).

The inhibitory effect of the tested treatments was depicted from the lowest concentration of 50 µg mL^−1^ after 7 days (Figure 4A). Besides that, the macroscopic investigation revealed that the phytotoxic effect was markedly increased in the following order: Compounds **6** < **7** < **2** < EtOAc extract < **4** < **3** < **5** < **1**, but without any morphological change neither on the roots nor on shoots. In addition, the microscopic investigation was carried out in the presence of EtOAc extracts of *C. arabica* and its identified compounds (**1**–**7**) tested the lowest concentration of 50 µg mL^−1^ on lettuce seeds for 48 h at (Figure 4B). The inhibitory effect of the extract and the identified compounds were further confirmed by microscopic studies involving determination of mitotic index reduction. The results showed that the compounds **1** and **5** reduced significantly the cell division through a marked decline in the mitotic index of 75.5% and 68.5%, respectively. It was noted also that the EtOAc extract and compounds **3** and **4** decreased the mitotic index by an inhibition of 51.6%, while this reduction did not exceed 30% for the remained treatments.

According to our previous results [3], the mito-depressive effect could be attributed to the phytotoxic isolated compounds from silique of *C. arabica.* Thus, the present study revealed, the identified allelochemicals, compounds **1** and **5**, were considered the major depressor of the mitotic index compared to the other identified compounds. In fact, the mitotic index measures the proportion of cells in the M-phase of the cell cycle and its inhibition could be explained as cellular death or a delay in the cell proliferation kinetics [25]. It was noted that some allelochemicals affected the mitotic process, particularly the G_2_-M checkpoint of lettuce, and reduced the number of cells in each cell division period [26,27]. Subsequently, they could affect the DNA synthesis by damaging the tubulins and resulting in polyploid nuclei [26,27,28]. Moreover, the phytotoxic action of the identified compound could be explained the influence of numerous physiological and metabolic processes.

### 2.4. Oxidative Stress Markers

#### 2.4.1. Membrane Integrity

The membrane damage in lettuce under the EtOAc silique extract and its identified compounds was estimated through relative electrolyte leakage (EL) and malondialdehyde (MDA) production (Table 3). The degree of cell membrane injury was perceived by an increase of the electrolyte leakage and malondialdehyde in whole plant of lettuce under all treatment cases. This obvious stimulation was markedly revealed by an average increase of 38.8% in lettuce under the compounds **1**, **3**, and **5**. The EL and MDA level were induced by an increment of 17.6% and 26.9%, respectively, under EtOAc extract, compounds **2** and **4**, but did not exceed 7.9% when the plant treated by the compound **7**. However, the compound **6** reduced the amount of major lipid peroxidation products and electrolyte leakage level in lettuce by an average decrease of 15.7%. The reduction of MDA level suggesting that the plant tissues of lettuce had the ability to maintain their membranes integrity.

#### 2.4.2. Hydrogen Peroxide (H_2_O_2_) Content

According to our previous studied [3,14] we have speculated that the inhibitory effect of the *C. arabica* extracts could be mediated induction of oxidative stress and ROS generation in target species. To address this possibility, we next determined the extent of ROS generation quantitatively in terms of hydrogen peroxide content in lettuce plant under *C. arabica* extract and its identified compounds. Overall, the results showed a great sensitivity of lettuce cell membranes to allelochemicals stress present in *C. arabica* extract compared to control. As shown in Table 3, among the treatments, the compounds **1**, **3**, and **5** raised the H_2_O_2_ content in lettuce by an average increase of 3.28-fold. The other treatments enhanced the H_2_O_2_ content in lettuce by an average level of 11.63 nmoL g^−1^ FW, with the exception of the compounds **6** and **7**, which they induced the lowest accumulation of H_2_O_2_ by an average level of 5.65 nmoL g^−1^ FW compared to the control 4.6 nmol g^−1^ FW. 

The measurement of the oxidative markers (increment of MDA and H_2_O_2_ level) of treated lettuce showed that our extract and its identified compounds cause an oxidative stress that could initiate a sequence of reactions inducing damages in cellular organelles, ultimately leading to cell death [29]. Among all the tested compounds, the electrolyte leakage, the MDA and the H_2_O_2_ level were significantly increased in lettuce plant where they reached the maximum by the compounds **1**, **3**, and **5** resulting in disruption of membrane integrity. It was noted previously that the membrane functions and lipid stability were have deleterious effects on plants, and some natural products acts by interfering with the integrity of membranes, which can be evaluated by relative electrolyte leakage and MDA production. A decrease in membrane permeability could be due to peroxidation of polyunsaturated fatty acids in the membranes resulting in the formation of several by products, including malondialdehyde (MDA) [30]. Many studies have shown that membrane perturbations are often suggested the primary site of action of many allelochemicals that trigger further modifications in physiological processes of plant cell. At cellular level, it induces lipid peroxidation, affects some enzymatic activities and rapidly depolarizes the root cell membrane thereby increasing the membrane permeability, thus blocking plant nutrients uptake [31]. Moreover, the high production of H_2_O_2_ could interfere with the activity of enzymes, and therefore inhibit photosynthetic activity [32]. In the present study, the compounds from *C. arabica* induced an accumulation of H_2_O_2_ in lettuce that could be an important factor that regulates the occurrence of phytotoxicity in target species. Although evidence about allelochemical-induced oxidative stress together with increased activity of antioxidant enzymes is emerging [33,34], but, little information is available about the mechanisms by which allelochemicals induce the oxidative damage. To date, the mode of action of *C. arabica* and its identified allelochemicals have remained elusive. Several action modes of different identified allelochemicals from other species have been previously suggested, including direct inhibition of photosynthesis process and ion uptake, interruption of dark respiration, and ATP synthesis and ROS-mediated allelopathic mechanisms [35]. According to the previous study [36,37], some identified flavonoids could inhibit electron transport in Photosystem II (PSII) and reduce the enzymatic activity of plastoquinone. Thus, the high bioactivity of the identified compound from *C. arabica* can be linked to their ability to interact with membranes. To determine if this phytotoxic effect occurs through oxidative stress, we continue analyzing the activity of some antioxidant enzymes involved in the detoxification and balance of H_2_O_2_ level, as well as membrane damage.

### 2.5. Activation of Antioxidant Defensive Enzymes in Lettuce

**Figure 5:**
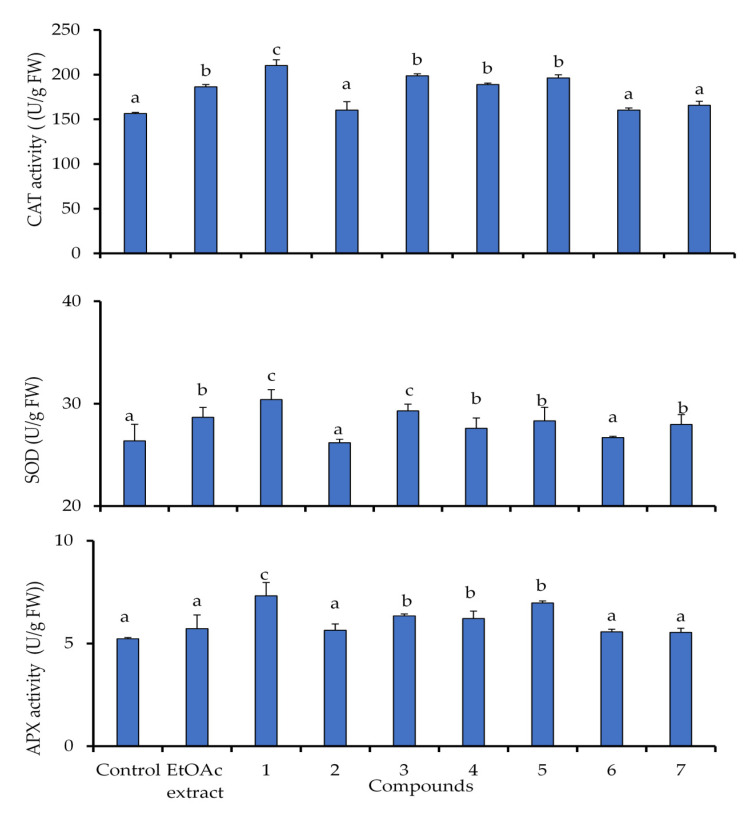
Superoxide dismutase (SOD), catalase (CAT), and ascorbate peroxidase (APX), activities in lettuce plants exposed to EtOAc extract of *C. arabica* and its identified compounds (**1**–**7**) at 50 µg mL^−1^. Values (*N* = 3 ± S.E.). Different letters in columns indicate significant differences among treatments at *p* < 0.05 (LSD test).

Plants respond to oxidative stress and ROS generation via the rapid stimulation of enzymatic antioxidant defense components. In this study, the antioxidant enzyme activities, including SOD, CAT and APX, were significantly increased in lettuce seedlings under the identified allelochemicals from *C. arabica* compared to untreated seedlings (Figure 5). These enzymes were more prominently influenced by compound **1** than the other treatments, and thus were consistent with their phytotoxic potential.

The activity of catalase (CAT) was increased in lettuce by 34.3% under the treatment of compound **1**, while the other compounds induced an average stimulation of 23.1%, except for the compounds **2**, **6** and **7** which they revealed the lowest stimulation of 6%. Similarly, superoxide dismutase (SOD) was induced by a slight stimulation of 15.2% under compound **1**, but this increase did not exceed 11.1% under the other treatments. The activities of ascorbate peroxidase (APX) was enhanced by 36.6% in lettuce seedlings under the compounds **1** and **5**. The induced stress response by compounds **3** and **4** was also depicted in lettuce by an average stimulation of 20%, as well as lesser activation was revealed by 7.4% under the other treatments (Figure 5).

Generally, the excessive production of antioxidant enzymes in treated plant under allelochemicals stress have evolved the lettuce a complex system of enzymatic antioxidant in order to reduce the induced oxidative. From our study, the compound **1** improved the antioxidant activity by increasing the enzymes compared to the other treatments. Stimulation of antioxidant activity is commonly associated with enhanced stress tolerance in plants. The activation of the antioxidant enzymes under allelochemical stress have been proved previously in other plant species [38,39]. Moreover, Allelochemical compounds can also cause oxidative damage and activate antioxidant mechanisms [40,41]. Our results are in accordance with the previous studies, some identified allelochemicals activated the CAT activity in maize seedlings [42], and cucumber cotyledons [43]. In addition, the enhanced activity of SOD indicated that excessive generation of O_2_^•−^ has been triggered by allelochemicals, which was then upregulated to mitigate the oxidative damage. However, these results disagree with other findings, which they have described the reduction of SOD activity in seeds of rape, cucumber, corn, sorghum [44] and lettuce under allelochemical stress [45].

### 2.6. Correlation between Phytotoxic Potential and Antioxidant Parameters

The detailed relationships between the physiological parameters, the oxidative stress markers and antioxidant enzymes defense system in lettuce plant treated with EtOAc extract and its identified compounds (Table 4). The results exposed strong positive correlation between growth parameters (RL and SL) and germination index that could demonstrate the strong inhibitory effect of the compounds. This inhibitory effect as explained by the great positive correlation between the MI and growth parameters, which was highly correlated with RL (*r* = 0.92, *p* < 0.01). However, the MI was negatively correlated with the oxidative stress markers and the antioxidant enzymes. It was noted that the oxidative stress markers (EL, MDA and H_2_O_2_) was positively correlated with antioxidant enzymes and was markedly positively correlated between H_2_O_2_ and CAT (*r* = 0.92, *p* < 0.01). According to Sánchez-Moreiras et al. [27], the negative effects on plant growth was highly correlated with a drastic inhibition of the mitotic activity. It has been demonstrated also that electrolyte leakage measurements may be correlated with several physiological and biochemical parameters conditioning the plant responses to environmental conditions [46].

### 2.7. Cluster and Principal Compound Analysis

**Figure 6:**
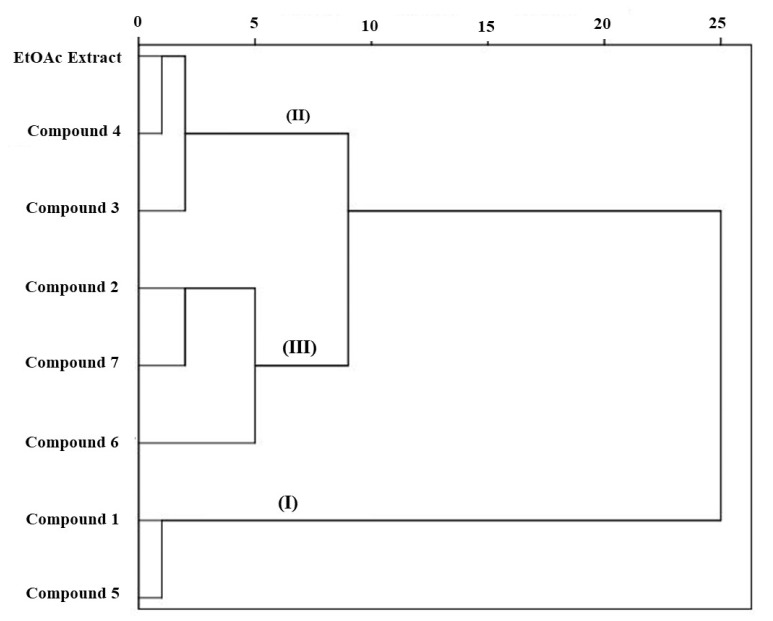
Cluster analysis of EtOAc extract of *C. arabica* silique and its identified compounds (**1–7**) based on their phytotoxic and antioxidant responses of lettuce plant.

Cluster analysis (CA) was conducted based on the physiological and antioxidant enzymes activities of the identified compounds from *C. arabica* silique EtOAc extract (Figure 6). This analysis revealed that the compounds are grouped into three clusters. This indicates that the identified compounds have different behavior based on the phytotoxic effect and antioxidant defense system in lettuce plant. It can also be seen that the compounds **1** and **5** are grouped in which are the most active compounds, while the lowest activities was exhibited by the third cluster grouping the compounds **2**, **6**, and **7**. The medium activities was recorded similar by the compounds **3**, **4**, and EtOAc extract which are clustered by the second group. This grouping gives indication that identified compounds in each group have different characteristics conducting to dissimilar effects among them.

**Figure 7:**
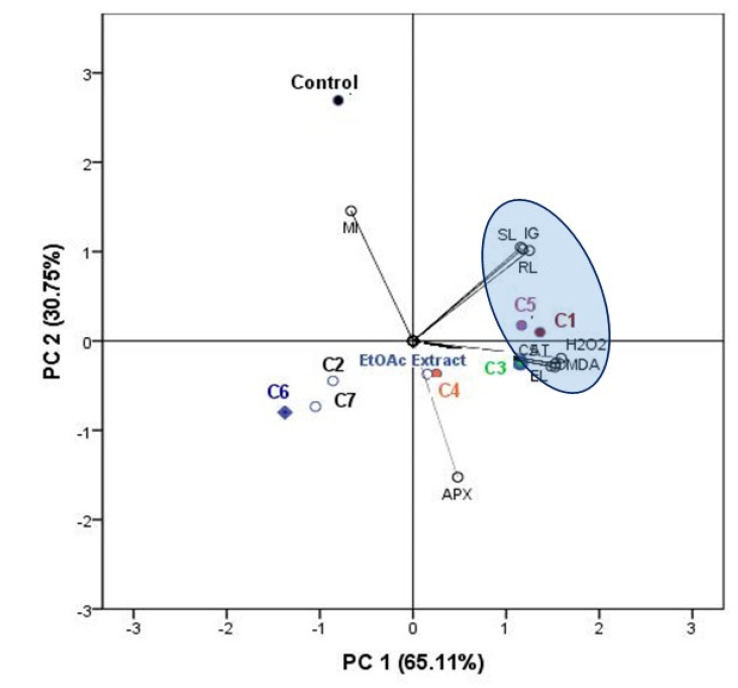
Biplot of the principal components analysis (PCA) based on the phytotoxic and antioxidant responses of lettuce plant to EtOAc extract of *C. arabica* silique and its identified compounds (**1–7**).

To obtain a more comprehensive understanding of the physiological responses of lettuce to different compounds, the results of PCA were presented by a biplot (Figure 7). The first two principal components explained 65.11% and 30.75% of the data variability, respectively. Almost all physiological and antioxidant parameters, including MDA, EL, H_2_O_2_, SOD, and CAT, were positively related to PC1, while the APX were negatively related to PC1. While, the MI was positively related to PC2. The compounds **1** and **5** exhibited great phytotoxic and antioxidant effects with an important damage in membrane integrity. It was noted that the EtOAc extract and the compound **4** are characterized by the same effects. However, the compounds **2**, **6**, and **7** have the weakest effect compared to the other treatments. While, the control MI has the greatest values compared to the other treatments (Figure 7). 

## 3. Materials and Methods

### 3.1. Plant Material

*C. arabica* was collected from the South-West of Tunisia (latitude 34°25’ N; longitude 8°46’ E). The plant was identified according to Tunisia flora [47] and authenticated by Dr. Afef Ladhari. A voucher specimen was dried and deposited in the herbarium of the university of Carthage, Tunisia.

### 3.2. Isolation and Identification of Bioactive Compound 

#### 3.2.1. General Experimental Procedures

^1^H and ^13^C NMR spectra were run on a Varian INOVA 500 NMR spectrometer at 500 and 125 MHz, respectively, in CDCl_3_ or CD_3_OD at 25 °C. UV–Vis spectra were recorded with a Varian Cary 300 UV–Vis spectrophotometer. LC-MS analyses were run on an Agilent LC-MS ESI-TOF 1260/6230DA instrument operating in positive ionization mode. Flash column chromatography was performed on Merck Kieselgel 60 (230–400 mesh) at a medium pressure. Column chromatography (CC) was performed on Merck Kieselgel 60 (70–240 mesh), on Sephadex LH-20 (Pharmacia). Analytical TLC was performed on Merck Kieselgel 60 F254 or RP-18 F254 plates with 0.2 mm film thickness. Spots were visualized by UV light or by spraying with EtOH:H_2_SO_4_ (93:7) followed by heating for 5 min at 110 °C. Preparative TLC was performed on Merck Kieselgel 60 F254 plates, with 0.5 or 1 mm film thickness. HPLC purifications were carried out on an Agilent 1100 HPLC system, equipped with an UV detector set at 280 nm. 

#### 3.2.2. Extraction and Isolation

Dried powder (1.5 kg) of silique of *C. arabica* was extracted twice at room temperature with MeOH during 48 h. After filtering, extracts were combined and dried at reduced pressure; the obtained residues were re-dissolved in MeOH: H_2_O (1:1) defatted with petroleum ether and extracted three times with ethyl acetate. Crude extract was dried with Na_2_SO_4_ and evaporated under reduced pressure originating a brown oil residue (97.3 g) that was purified by silica gel column chromatography (CC) using CH_2_Cl_2_ →EtOAc →MeOH →CH_2_Cl_2_ (2:1)→MeOH as eluents, to give 23 homogeneous fractions (AC1–AC23). Fraction AC9 (500.2 mg), eluted with MeOH:CH_2_Cl_2_ (2:1), was again purified on Sephadex LH-20 (CC) eluted with: *n*-hexane:CH_2_Cl_2_:MeOH (7:4:0.5→6:4:1)→MeOH, to give 16 homogeneous fractions (AC9_-1__AC9_-16_). AC9_-6_ fraction was further purified by preparative TLC (1 mm) on silica gel eluted with CH_2_Cl_2_:MeOH (9:1) obtained compound **5** (30.5 mg). The fraction AC9-13 (12.8 mg) was subjected to RP-18 HPLC with a mobile phase of MeCN:MeOH:H_2_O (4:5:1) to yield compound **1** (9.2 mg). Fraction AC17 (600.9 mg) of first CC was chromatographed by Sephadex LH-20 CC (MeOH→MeOH:H_2_O (8:2)) to give 2 fractions: AC17_-A_ (100.8 mg) and AC17_-B_ (450.6 mg). The fraction eluted with MeOH (AC17_-A_) was further purified on Sephadex LH-20 CC (MeOH:H_2_O (8:2)) to give 18 fractions (AC17-_A1-18_). The fraction AC17-_A12_ was purified by RP-18 HPLC with a mobile phase MeCN:MeOH: H_2_O (3:6:1) to obtain the compound **3** (9.7 mg). AC17_-B_ was purified on Sephadex LH-20 CC with MeOH:H_2_O (8:2) as eluent, given 9 fractions (AC17-_B1-9_). AC17_-B-6_ was again purified on Sep-Pak C-18 eluted with H_2_O→MeOH (1:1)→MeOH to yield compound **4** (10 mg). The fraction AC4 (668.3 mg) of first CC, was further fractionated by flash column chromatography on silica gel eluted with EtOAc:CH_2_Cl_2_ (1:9→1:4→ 3:7)→MeOH to yield 10 homogeneous fractions (AC4A-J). The fraction AC4A (350.6 mg) was next fractionated by flash column chromatography on silica gel eluted with EtOAc:CH_2_Cl_2_ (1:9→1:4→3:7)→EtOAc→ MeOH: EtOAc (1:9)→MeOH to get 17 fractions (AC4_A-1-17_). The fraction AC4_A-8_ eluted with EtOAc:CH_2_Cl_2_ (1:4) was subjected to RP-18 HPLC with CH_3_CN:MeOH:H_2_O (2:5:3) as mobile phase to obtain the compound **2**. Finally, AC4E (18 mg) was purified by RP-18 HPLC (CH_3_CN:MeOH:H_2_O (2:5:3)) to obtain compounds **6** and **7**.

### 3.3. Phytotoxic Assays

The phytotoxic activity was achieved through the assessment of the ethyl acetate extract from *C. arabica* silique and its identified compounds on the germination and seedling growth of *L. sativa.* The seeds were surface sterilized with sodium hypochlorite solution (0.4%, *v/v*) for 3 min and soaked in sterile distilled water for 30 min. Twenty seeds were sown in Petri dishes containing layers of Whatman filter paper, impregnated with 5 mL of distilled water (control) or 5 mL of tested solution. The ethyl acetate extract and its identified compounds were diluted with methanol to the desired concentrations (50, 100, 200, 400, 800 µg mL^−1^). Two sets of Petri plates were prepared. In the first set, imbibed seeds were used to evaluate the effect of extracts on germination. The second set of pre-germinated seeds with 1 mm root length, was used to evaluate the effect of the extract and its isolated compounds on root and shoot lengths. The Petri dishes were placed in a growth chamber at 24/22 °C for 14/10 h light and dark periods, respectively. Germination was determined by counting the number of seeds that had germinated at 24 h intervals over 6 days. Shoot and root lengths were measured 7 days after placing the pre-germinated seeds in each Petri dish. Data were transformed to percent of control for analysis. The index of germination (GI) and the % inhibition/stimulation were calculated according to Chiapuso et al. [48] and Chung et al. [49], respectively.

### 3.4. Cytogenotoxicity Test

The cytogenotoxicity assays of *C. arabica* ethyl acetate extract and its identified compounds were assessed by the same environmental conditions as those previously described. Root tips were collected 24 h after the start of the experiment, when the newly emerged roots reached 1.50–2.00 cm in length, they were used. The newly emerged roots were treated by the lowest concentration of the acetate extract and the identified compounds at 50 µg mL^−1^ for 48h. To avoid toxic effect of solvents, the filter papers were placed in a fume hood for 30 min to allow complete solvent evaporation. Subsequently, 5 mL of distilled water were added to each Petri dish. The control group was treated with distilled water. At the end of each exposure period, root tips were cut and subsequently fixed in Carnoy’s solution (absolute ethanol/glacial acetic acid (3:1, *v/v*)) for 24 h and stored at −18 °C. The cytological preparations were made using the method described previously by Koodkaew et al. [50] with some modifications. Staining of the chromosome was carried out with acetic carmine for 30 min. One mm of the meristematic zone was immersed in a drop of 45% acetic acid on a clean slide and squashed under a cover slip by thumb pressure. For each treatment, 1000 cells were evaluated to determine the mitotic index (MI). The mitotic index (MI) was calculated as the proportion of dividing cells (M phase) to the total number of cells observed. The frequency of each mitotic phase was calculated as the percentage in relation to the number of cells in mitosis in the treatment.

### 3.5. Oxidative Stress Response of Lettuce to C. arabica Extract and Its Identified Active Compounds

The Oxidative stress markers and the antioxidant enzyme activity of *C. arabica* ethyl acetate extract and its identified compounds were investigated on lettuce at the lowest concentration of 50 µg mL^−1^. The seeds of lettuce were grown for 10 days under the same conditions described for the previous phytotoxic assays, in order to obtain the biomass needed for extraction.

#### 3.5.1. Determination of Electrolyte Leakage (EL) 

According to Shalata and Neumann [51] the electrolyte leakage (EL) was estimated. The control and treated fresh lettuce were homogenized in tubes containing 25 mL of distilled water at room temperature in dark. After 24 h, the initial bathing solution electrical conductivity (L1), where the samples were immersed, was measured using a digital conductivity meter (type BCT-4308). The samples were then autoclaved at 121 °C for 20 min in order to burst cell walls and liberate all electrolytes, and then cooled down to 25 °C, there after incubated again in distilled water as indicated previously. After 24 h, a last conductivity reading (L2) was obtained. The electrolyte leakage (EL) was defined as follows:

EL (%) = (L1/L2) × 100.(1)

#### 3.5.2. Lipid Peroxidation 

The malondialdehyde (MDA) is produced during lipid peroxidation and can be used as a marker of oxidative stress. Its concentration in the whole lettuce plant was measured three days after the treatments [52]. The samples of 250 mg were homogenized with a mortar and pestle in 2.5 mL of a mixture containing phosphate buffer 67 mM and 500 mg of PVP (polyvinylpyrrolidone). The mixture was centrifuged at 2000 rpm for 15 min at 4 °C. An assay mixture containing 2 mL of the supernatant and 2 mL of 0.5% (*w*/*v*) thiobarbituric acid (TBA) in 20% (*w*/*v*) trichloroacetic acid (TCA) was heated at 90 °C for 10 min and then rapidly cooled in an ice bath. After centrifugation (2,000 g for 10 min at 4 °C), the supernatant absorbance was recorded at 532 nm and corrected for non-specific absorbance at 600 nm. Lipid peroxidation products were measured as the content of TBA-reactive substances. Its content (nmol g^−1^) was calculated according to the molar extinction coefficient of 155 mM^−1^ cm^−1^.

#### 3.5.3. Determination of Hydrogen Peroxide (H_2_O_2_) Content

Hydrogen peroxide (H_2_O_2_) is produced during oxidative stress. Its content in the lettuce plants was determined using the protocol described by Velikova et al. [53]. Treated plants were extracted with trichloroacetic acid mL of 0.1 % TCA in an ice bath, and centrifuged at 12,000 rpm for 15 min. Then, 0.5 mL of supernatant was mixed with 0.5 mL phosphate buffer (pH 7) and 1 mL of potassium iodide (1 M). The absorbance of this reaction mixture was measured at 390 nm. Hydrogen peroxide content was determined using the extinction coefficient 0.28 μM^−1^ cm^−1^ and expressed as nmol g^−1^. 

### 3.6. Antioxidant Enzyme Activities

To extract antioxidant enzymes, whole lettuce plant (200 mg) (from each treatment or control) was homogenized in 10 mL of sodium phosphate buffer (0.1 M, pH 7.0). The homogenized material was centrifuged at 13,000 rpm for 30 min at 4 °C, and the supernatant was collected for the enzymatic analyses of superoxide dismutase (SOD), catalase (CAT), and ascorbate peroxidase (APX), which was quantified from spectrophotometer readings. The final volume of the reaction for reading enzyme activity was 2 mL. All readings were conducted in triplicate.

Superoxide dismutase was assayed according to the following method proposed by Giannopolitis and Ries [54]. The absorbance was determined at 560 nm and one unit of SOD activity defined as the quantity of the enzyme that inhibits nitro-blue tetrazolium (NBT) photoreduction by 50%. Catalase activity was measured as per the method of Cakmak and Marschner [55]. The reaction mixture (2 mL) consisted of 25 mM phosphate buffer (pH 7.0), 10 mM H_2_O_2_ and 0.2 mL of enzyme extract. The activity was determined by measuring the rate of disappearance of H_2_O_2_ for 1 min at 240 nm and calculated using an extinction coefficient of 39.4 mM^−1^ cm^−1^ and expressed as enzyme units g^−1^ FW. One unit of CAT activity was defined as the amount of enzyme that catalyzes decomposition of 1 µM min^−1^ of H_2_O_2_. The ascorbate reductase activity was quantified according to the following method proposed by Nakano and Asada [56] using an extinction coefficient of 2.8 mM^−1^ cm^−1^ by measuring the decrease in absorbance at 290 nm for 1 min. It was expressed as enzyme units g^−1^ FW. One enzyme unit was defined as the amount of enzyme required to oxidize 1 μM of ascorbate min^−1^.

### 3.7. Statistical Analyses

The laboratory bioassays in a complete randomized design with three replications were performed using IBM SPSS Statistics 20.0, to evaluate the effects of *C. arabica* extracts and its identified compounds over the control values. Experimental data were subjected to one-way analysis of variance (ANOVA) and a post hoc LSD tests, to determine significance differences among mean values at the probability level of 0.05. The data obtained for all parameters in accordance with all tested methanol extracts were subjected to principal components analysis (PCA) and hierarchical cluster analysis (HCA) using SPSS 20.0 software.

## 4. Conclusions

This is the first report on the mode of action of the identified allelochemicals from the silique of *C. arabica*. The allelochemical action was mainly evident from the lowest concentration of 50 µg mL^−1^, which interfered with the initial growth of *L. sativa* seedlings. Cleomoside A was responsible for the most phytotoxic effect, promoting a drastic reduction in root, and shoot lengths, as well as frequency of germinated seeds. This compound influenced the cell cycle by reducing the mitotic index, indicating its cytotoxicity. Furthermore, the phytotoxic effects of *C. arabica* based compounds are positively and negatively correlated with oxidative markers and plant antioxidant enzymes (SOD, APX, and CAT) respectively, which contribute to the defence mechanism. The physiological and cytological changes caused by the identified compound, described here, help to get an insight into the detailed mode of action of allelochemical such as flavonoids, triterpene and thiohydroximate. This study provides the first information about the possible exploitation of the identified compounds from *C. arabica*, especially cleomoside A: A thiohydroximate, as a new bioherbicide, since it probably acts by inhibiting or disturbing cell division. 

## Figures and Tables

**Table 1:** ^1^H and ^13^C NMR spectral data of **1** (in methanol-*d*4, ^1^H: 500 MHz, ^13^C: 125 MHz.

No.	1		
	δC (mult.)	δH ^a^	HMBC Correlations
SMe	12.6 (q)	2.24 s	0
0	171.0 (s)		
1	39.0 (t)	4.78, d (14.4) 4.50, d (14.4)	0, 2, 3, 9
2	124.7 (d)	7.10, s	1, 3, 8, 9
3	113.5 (s)		
4	107.8 (d)	7.02 (overlapped)	5, 8, 9
5	153.5 (s)		
6	104.9 (d)	6.80, br d (7.1)	4, 7, 8
7	123.9 (d)	7.02 (overlapped)	5, 8
8	140.5 (s)		
9	119.0 (s)		
1′	102.5 (d)	5.10, d (7.1)	5, 3′
2′	75.8 (d)	3.53, m	4′
3′	78.8 (d)	3.44, m	5′
4′	72.0 (d)	3.42, m	6′
5′	78.8 (d)	3.61, m	1′
6′	63.1 (t)	3.92, dd (13.5, 10.2) 3.75, dd (12.0, 5.4)	5′

^a^ Coupling constants are given in parentheses, *J* in Hz.

**Table 2:** Effect of silique ethyl acetate extract of *C. arabica* and its identified compounds (C1–C7) on germination index, for seven days, expressed in % of control of lettuce.

	Concentrations (µg mL^−1^)						
	Treatments	50	100	200	400	800	IC_50_
	**EtOAc Extract**	89.3 ± 3.4 ^c^	77.6 ± 1.5 ^c^	70.7 ± 4.9 ^c^	69.6 ± 1.3 ^c^	71.9 ± 3.6 ^b^	>800
**Compounds**	**1**	26.3 ± 2.1 ^a^	25.1 ± 1.7 ^a^	15.3 ± 0.9 ^a^	00.0 ± 0.0 ^a^	00.0 ± 0.0 ^a^	<50
	**2**	93.2 ± 1.4 ^c^	90.7 ± 5.6 ^d^	89.6 ± 1.2 ^d^	91.3 ± 2.6 ^d^	70.4 ± 5.1 ^b^	>800
	**3**	86.3 ± 4.3 ^c^	80.4 ± 4.7 ^c^	78.7 ± 2.7 ^c^	66.3 ± 3.6 ^c^	60.4 ± 3.9 ^b^	>800
	**4**	93.6 ± 2.9 ^c^	86.7 ± 4.3 ^c^	82.9 ± 3.9 ^d^	80.4 ± 3.4 ^d^	77.3 ± 5.3 ^b^	>800
	**5**	65.3 ± 3.4 ^b^	51.1 ± 3.6 ^b^	30.4 ± 1.6 ^b^	25.0 ± 2.3 ^b^	00.0 ± 0.0 ^a^	91.4
	**6**	96.3 ± 4.9 ^c^	94.2 ± 5.4 ^d^	94.6 ± 4.3 ^d^	88.2 ± 3.2 ^d^	80.4 ± 4.1 ^b^	>800
	**7**	93.4 ± 3.6 ^c^	93.7 ± 4.6 ^d^	91.9 ± 6.4 ^d^	90.7 ± 5.4 ^d^	90.7 ± 2.6 ^c^	>800

Note: The same letter indicates no significant differences *p* < 0.05 (LSD test).

**Table 3:** Changes in hydrogen peroxide (H_2_O_2_), electrolyte leakage (EL) and malondialdehyde (MDA) contents in lettuce plant grown under silique EtOAc extract of *C. arabica* and its identified compounds at 50 µg mL^−1^.

		EL (%)	MDA (nmol g^−1^ FW)	H_2_O_2_ (nmol g^−1^ FW)
	**Control**	150.6 ± 2.6 ^b^	50.3 ± 2.3 ^a^	4.6 ± 1.5 ^a^
	**EtOAc**	177.3 ± 5.1 b ^c^	64.2 ± 1.9 ^b^	12.4 ± 2.1 ^b^
**Compounds**	**1**	210.6 ± 3.9 ^c^	70.2 ± 4.5 ^c^	15.3 ± 2.6 ^c^
	**2**	170.3 ± 1.6 b ^c^	62.3 ± 2.4 ^b^	10.2 ± 2.4 ^b^
	**3**	201.6 ± 2.5 ^c^	71.6 ± 2.1 ^c^	14.9 ± 3.4 ^c^
	**4**	183.7 ± 2.6 b^c^	64.7 ± 3.7 ^b^	12.3 ± 1.9 ^b^
	**5**	200.9 ± 3.1 ^c^	72.6 ± 5.9 ^c^	15.1 ± 0.6 ^c^
	**6**	124.3 ± 0.9 ^a^	43.1 ± 3.1 ^a^	5.2 ± 0.5 ^a^
	**7**	154.9 ± 1.3 ^b^	54.3 ± 7.3 ^a^	6.1 ± 0.5 ^a^

Note: The same letter indicates no significant differences *p* < 0.05 (LSD test).

**Table 4:** Pearson correlations between analyzed phytotoxic parameters, oxidative stress markers and antioxidant enzymes responses of lettuce plants to allelochemical stress of *C. arabica.*

	GI	RL	SL	MI	EL	MDA	H_2_O_2_	CAT	SOD	APX
**IG**										
**RL**	0.920 **									
**SL**	0.929 **	0.947 **								
**MI**	0.699 *	0.924 **	0.843 **							
**EL**	0.433	0.631	0.494	−0.880 **						
**MDA**	0.322	0.528	0.377	−0.874 **	0.978 **					
**H_2_O_2_**	0.282	0.471	0.304	−0.951 **	0.947 **	0.962 **				
**CAT**	0.349	0.555	0.429	−0.946 **	0.900 **	0.854 **	0.922 **			
**SOD**	0.267	0.464	0.374	−0.813 **	0.765 *	0.690 *	0.747 *	0.907 **		
**APX**	0.514	0.570	0.518	−0.934 **	0.842 **	0.781 *	0.847 **	0.905 **	0.755 *	

Notes: **GI**, germination index; **RL**, root length; **SL**, shoot length; **MI**, Mitotic Index; **El**, Electrolyte Leakage; **MDA**, lipid peroxidation; **H_2_O_2_**, hydrogen peroxide; **CAT**, Catalase; **SOD**, superoxide dismutase; **APX**, ascorbate peroxidase. ** Correlation is significant at the 0.01 level. * Correlation is significant at the 0.05 level.

## Supplementary Materials

The following are available online, Figure S1: ^1^H NMR spectrum compound **1**, Figure S2: ^13^C NMR spectrum compound **1**, Figure S3: COSY spectrum compound **1**, Figure S4: HSQC spectrum compound **1**, Figure S5: HMBC spectrum compound **1**, Figure S6: DEPT spectrum compound **1**, Figure S7: Mass spectrum compound **1**.
