# Supplementary material for: Physiological and Oxidative Stress Responses of Lettuce to Cleomside A: A Thiohydroximate, as a New Allelochemical from Cleome arabica L
Source: Molecules. 2020 Sep 28;25(19):4461. doi: 10.3390/molecules25194461 (PMC7582273; doi:10.3390/molecules25194461)
Supplement: Supplementary file 1 [file molecules-25-04461-s001.pdf]

molecules

Physiological and Oxidative Stress Responses of Lettuce to Cleomside A: A Thiohydroximate, as a New Allelochemical from *Cleome arabica* L.

Afef Ladhari [1,2,*], Anna Andolfi [2,3] and Marina DellaGreca [2]

[1] Laboratoire GREEN-TEAM (LR17AGR01), Institut National Agronomique de Tunisie (INAT), Universite de Carthage, 43 avenue Charles Nicolle, 1082 Tunis, Tunisia
[2] Dipartimento di Scienze Chimiche, Università Federico II, Complesso Universitario Monte S. Angelo, via Cintia, 4, 80126 Napoli, Italy; andolfi@unina.it (A.A.); dellagre@unina.it (M.D.)
[3] BAT Center-Interuniversity Center for Studies on Bioinspired Agro-Environmental Technology, University of Napoli 'Federico II', 80138 Naples, Italy
* Correspondence: afef.ladh@yahoo.fr; Tel.: +216-222-13830

SUPPORTING INFORMATION

Contents:

1D and 2D NMR Spectra of compound **1** pages S-2 to S-7
Mass spectrum compound **1** page S-8

^{1}H NMR cleomside A

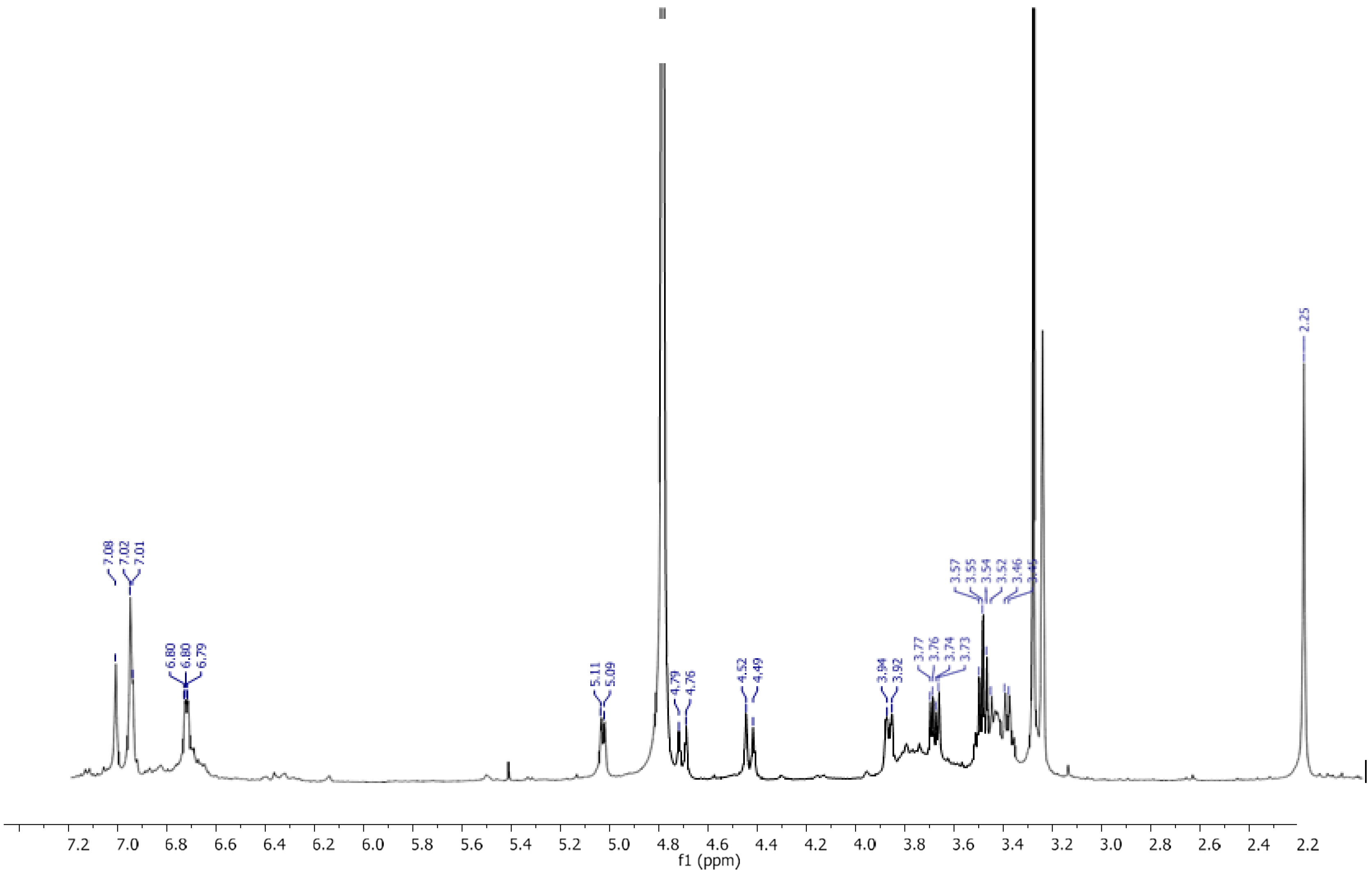

Figure S**1.** ^{1}H NMR spectrum compound **1**.

^{13}C NMR cleomside A

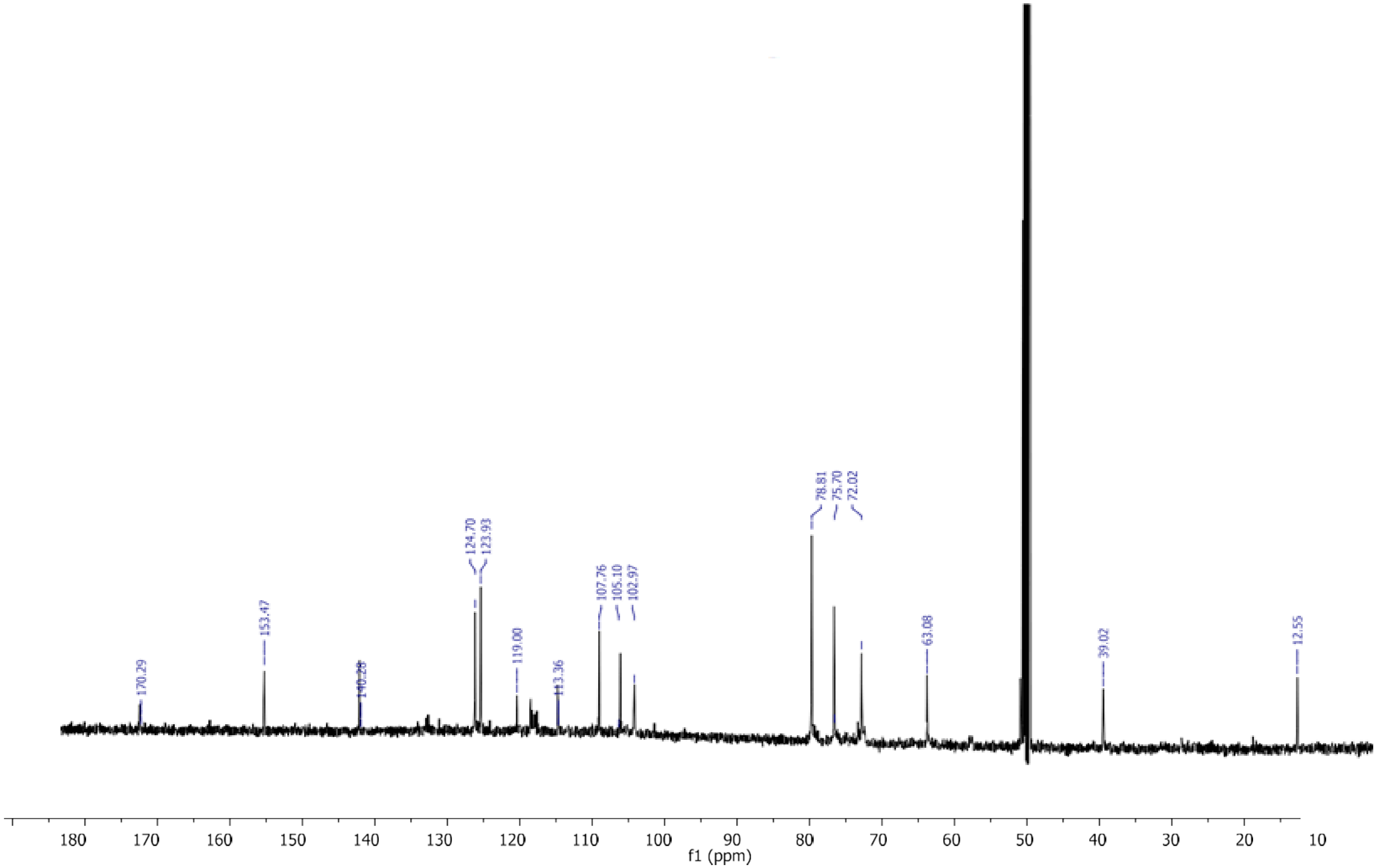

Figure S**2.** ^{13}C NMR spectrum compound **1**.

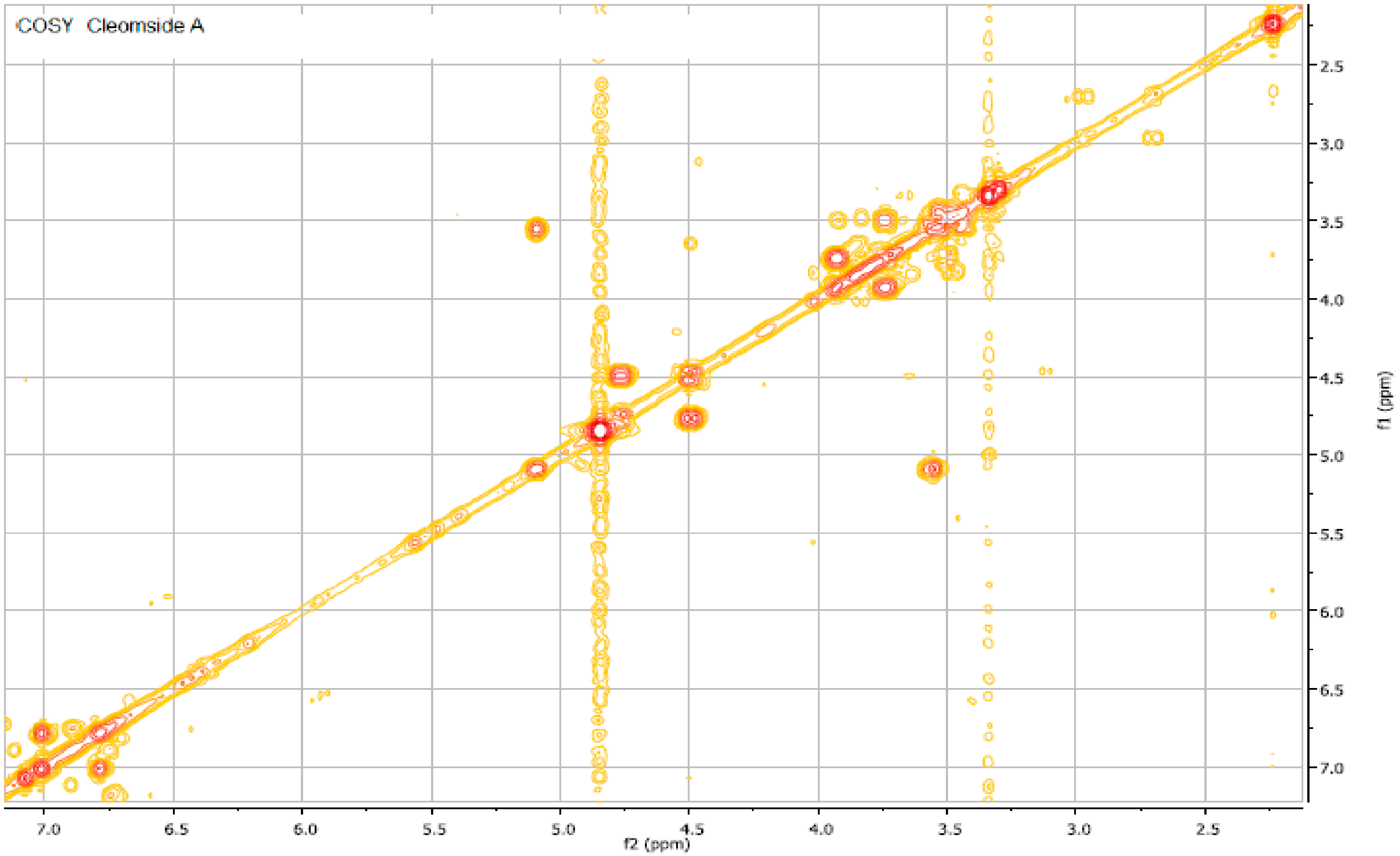

Figure S3**.** COSY spectrum compound **1**.

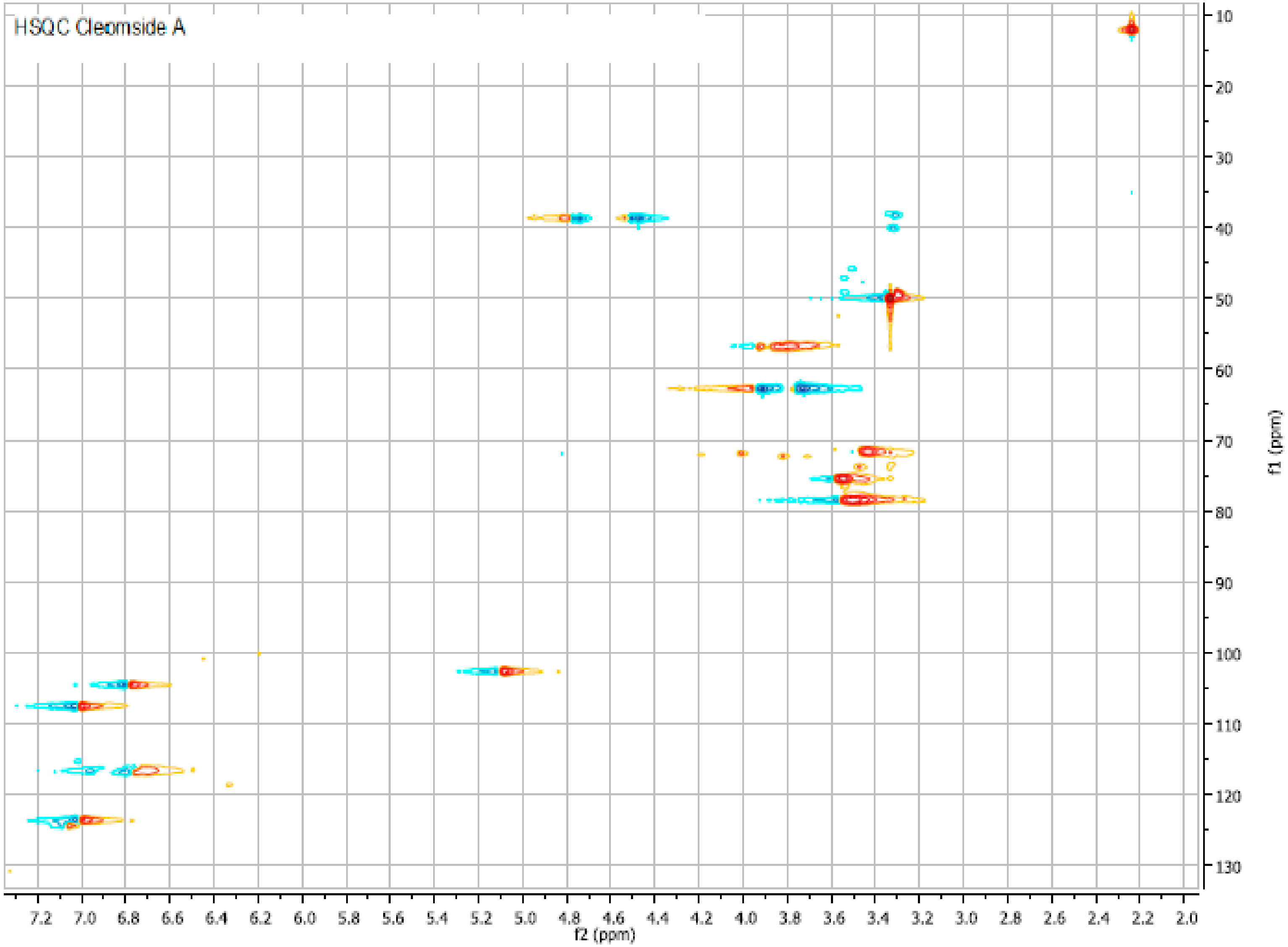

Figure S4**.** HSQC spectrum compound **1**.

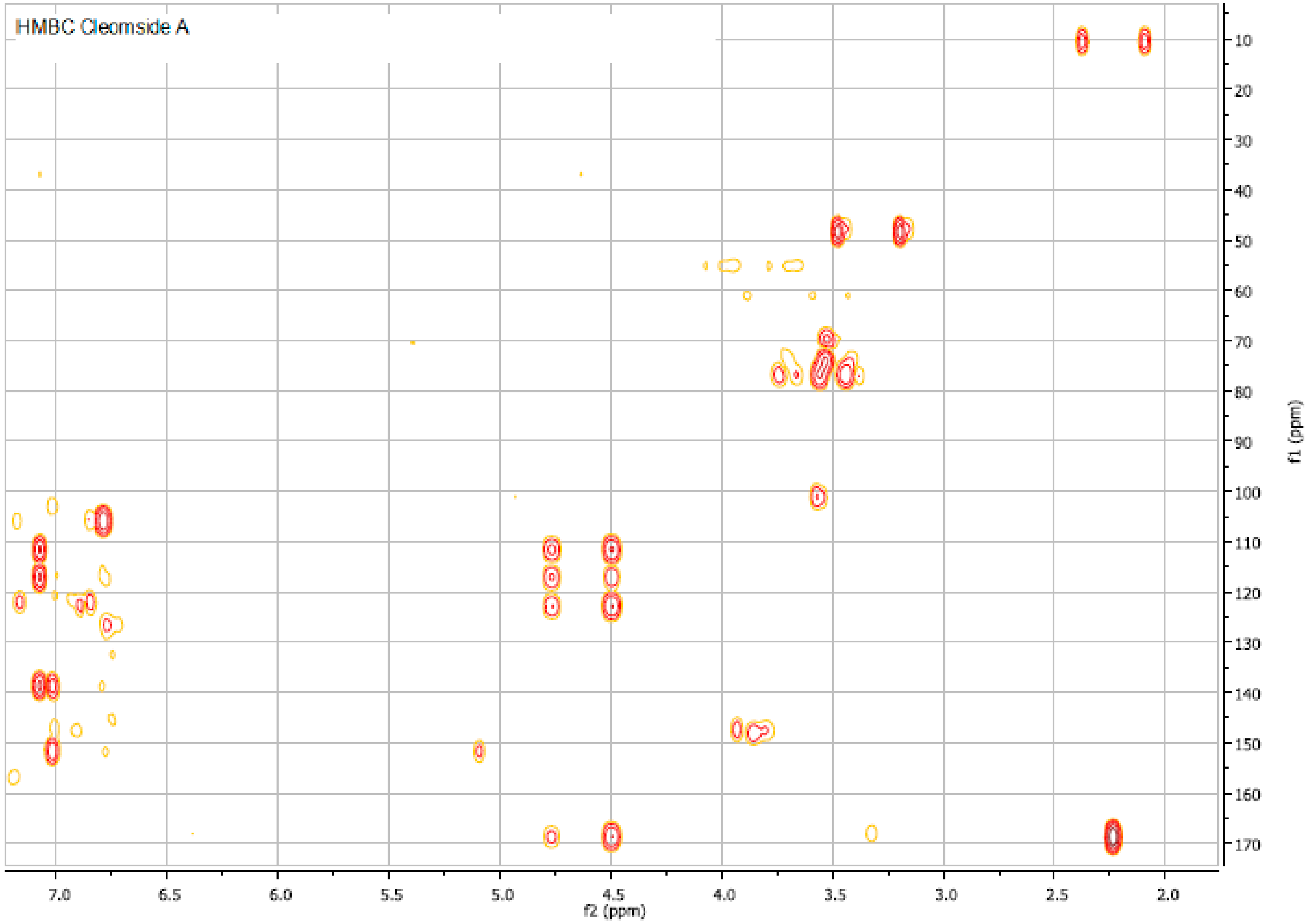

Figure S5. HMBC spectrum compound **1**.

DEPT Cleomside A

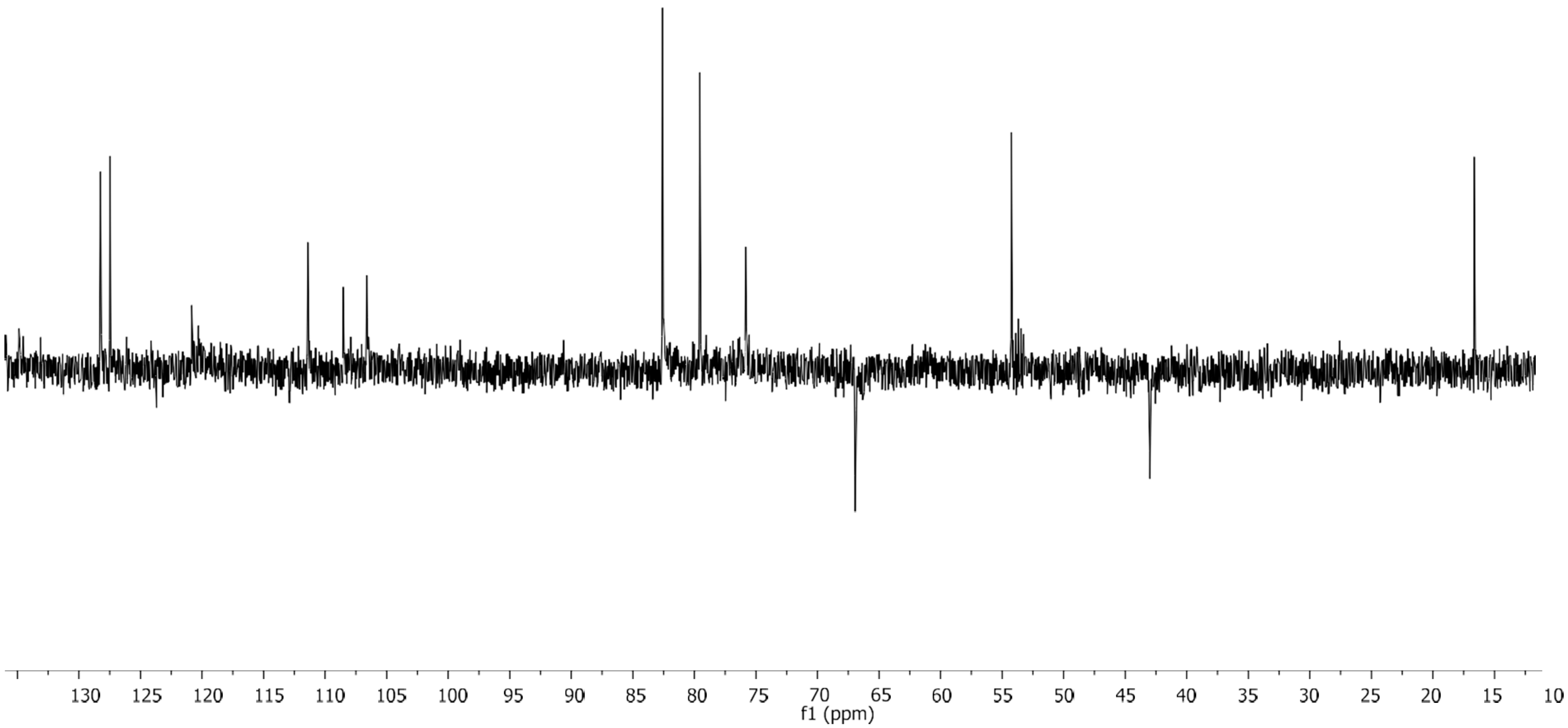

Figure S6. DEPT spectrum compound **1**.

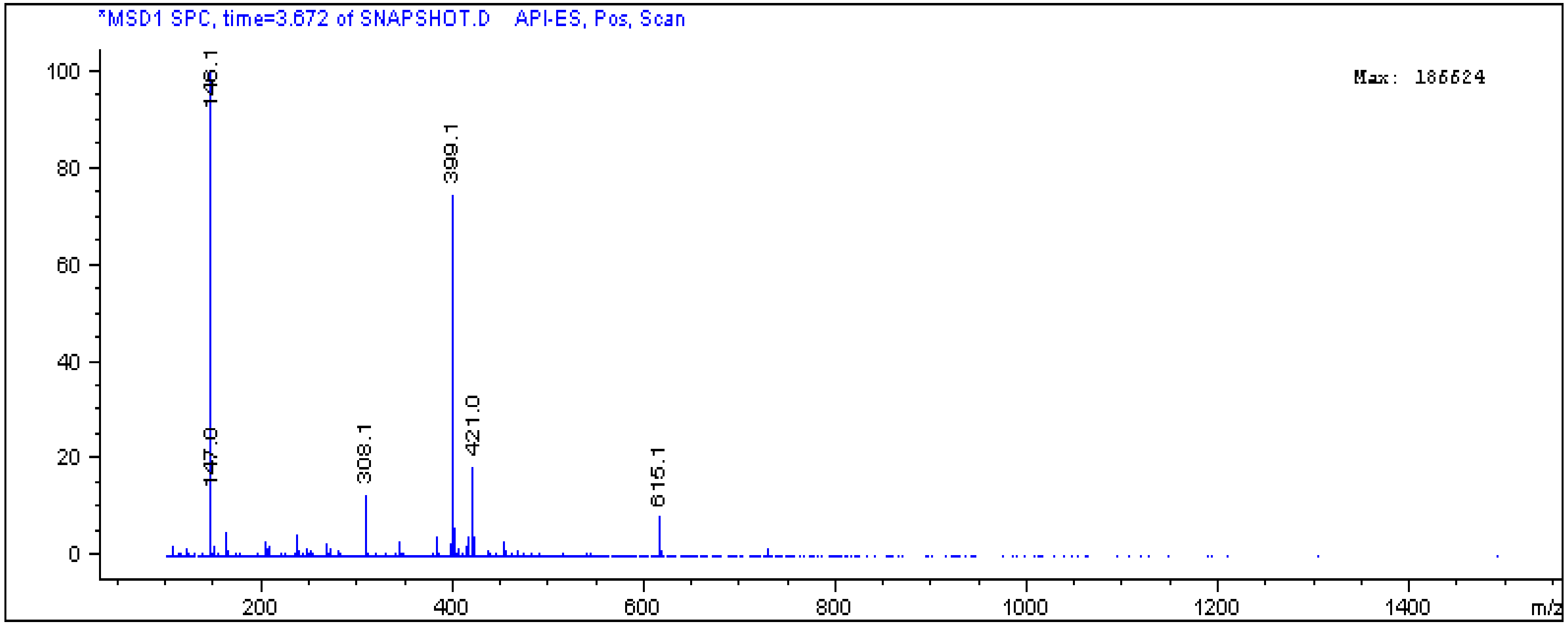

Figure S7. Mass spectrum compound **1**.
